# Lactoferrin Enhanced Apoptosis and Protected Against Thioacetamide-Induced Liver Fibrosis in Rats

**DOI:** 10.3889/oamjms.2015.038

**Published:** 2015-03-31

**Authors:** Alyaa Hessin, Rehab Hegazy, Azza Hassan, Nemat Yassin, Sanaa Kenawy

**Affiliations:** 1 *National Research Center, Medical Division, Pharmacology Department, Giza, Egypt*; 2 *Cairo University, Faculty of Veterinary Medicine, Pathology Department, Giza, Egypt*; 3 *Cairo University, Faculty of Pharmacy, Pharmacology and Toxicology Department, Cairo, Egypt*

**Keywords:** Liver fibrosis, Lactoferrin, Thioacetamide, Apoptosis, NF-κB, Alpha fetoprotein

## Abstract

**BACKGROUND:**

Liver fibrosis is the common pathologic consequence of all chronic liver diseases.

**AIM:**

Lactoferrin (Lf) was investigated for its possible hepatoprotective effect against thioacetamide (TAA)-induced liver fibrosis rat model.

**MATERIAL AND METHODS:**

Rats received TAA (200 mg/kg/biweekly, ip) for four successive weeks. Lf (200 mg/kg/day, p.o.) or vehicle (VHC) was administered for one month before and another month during TAA injection. Body weight and mortality rate were assessed during the month of TAA-intoxication. Thereafter, serum and liver tissues were analyzed for liver function, oxidative, fibrotic and apoptotic markers.

**RESULTS:**

Lf conserved rats against TAA-induced body weight-loss and mortality. Preservation of serum albumin, alkaline phosphatase and total bilirubin levels was also observed. Lf also protected rats against TAA-induced decrease in reduced glutathione and increase in malondialdehyde liver contents. Normal liver contents of hydroxyproline, nuclear factor kappa B and alpha fetoprotein; as markers of fibrosis; were increased with TAA and conserved with Lf-TAA. Lf maintained the normal architecture of the liver and immunohistochemical findings revealed increase in apoptotic bodies compared to TAA that favored necrosis.

**CONCLUSION:**

In conclusion, Lf improved liver function, reduced oxidative stress and liver fibrosis, and enhanced apoptosis in rats with liver fibrosis, suggesting it to have useful therapeutic potential in patients with liver fibrosis.

## Introduction

Liver fibrosis is a major global health problem causing approximately 1.4 million deaths per year [1]. It is the common pathologic consequence of all chronic liver diseases. Its principal causative factors in developing countries are viral infection with hepatitis B and C and parasitic infection with *Schistosoma Mansoni*, while it is excessive alcohol consumption in developed countries [2]. Some drugs, autoimmune diseases and genetic disorders are also contributed to liver fibrosis [3-5]. Persistent inflammation caused by chronic liver injury, resulted in aberrant wound healing response with excessive deposition of extracellular matrix (ECM) in the space of Disse that leads to disruption of normal microanatomy of liver sinusoids [6]. The principal cellular source of ECM has been reported to hepatic stellate cells (HSCs) [7], which activated from a quiescent, vitamin A storing cell to an activated myofibroblast-like phenotype under influence of inflammatory mediators and growth factors [8]. Once activated, HSCs up-regulate gene expression responsible for synthesis of ECM components, leading to deposition of collagen I and III, elastin and glycoproteins in the space of Disse [9]. Activated HSCs themselves secrete inflammatory mediators, a vicious cycle is formed, and a perpetual process of liver damage and repair leads to continuous deposition of ECM [10].

Liver fibrosis could be considered a bidirectional process and could be reversible [11]. The hope is that if antifibrotic therapy can reconstitute the normal balance of liver, normal function can be restored and clinical manifestations may regress. Current and evolving approaches primarily target to inhibit the activated HSCs, proliferation, and products as well as enhance their apoptosis [12].

Lactoferrin (Lf) is a natural glycoprotein found predominantly in milk [13]. It has multi-pharmacological properties that are mediated through specific receptors present on the surface of many cells [14, 15]. Lf is well known for its anti-bacterial, anti-fungal and anti-parasitic activities [15, 16]. It also has anti-viral effect against wide range of viruses including hepatitis C (HCV), human immunodeficiency, herpes simplex and cytomegalovirus [14, 17], and showed anti-carcinogenic effect against many tumor cells [18, 20].

In liver, Lf prevented hepatocellular necrosis and revealed a direct cytoprotective function through anti-oxidant activity [21]. Lf has been also found to inhibit HSCs activation through a potent anti-inflammatory effect [22]. In contrast, it favored HSCs apoptosis through induction of natural killer cells (NK), macrophages and CD8+ T-lymphocytes [23]. The role of Lf against liver fibrosis has not been elucidated yet. Nevertheless, a potential anti-fibrotic action may be returned to its anti-inflammatory, anti-oxidant and pro-apoptotic actions. The current study was conducted to evaluate this possible antifibrotic effect of Lf against liver fibrosis induced experimentally in rats using thioacetamide (TAA); a model that results in biochemical and histological changes similar to that of human liver fibrosis [24].

## Materials and Methods

### Animals

Adult male albino Wistar rats, weighing 200-250 g, were obtained from animal house colony of National Research Center (NRC) (Giza, Egypt), and treated according to ethics guidelines stated by the ethics committee of NRC.

### Drugs and chemicals

TAA was obtained from Loba Chemie (India) as creamy white crystals freely soluble in water, and freshly prepared as solution in saline (0.9% NaCl) for intraperitoneal (ip) injection in rats.

Lf was obtained from Radiance Nutritional Company (New Zealand) as pinkish white crystals slightly soluble in water, and freshly prepared as suspension in 1% (v/v) Tween 80 in distilled water for oral administration.

### Experimental Design

Thirty six rats were randomly allocated into three groups; the 1^st^ received vehicles (VHC) and served as normal (VHC group), while the 2^nd^ and 3^rd^ groups served as liver fibrosis-model group (TAA group) and Lf-treated group (Lf-TAA group), respectively. VHC group received saline ip twice weekly for four weeks. In the other two groups, liver fibrosis was induced using TAA (200 mg/kg/biweekly, ip) [25], on Sundays and Wednesdays, for four successive weeks. For one month before and another month during induction of liver fibrosis, rats of VHC (n = 6) and TAA (n = 18) groups received daily oral dose of 1% (v/v) tween 80 in distilled water, while rats of Lf-TAA group (n = 12) received Lf (200 mg/kg/day, p.o.) [26].

### Assessment of body weight change and mortality percent

Body weights of rats in each group were measured weekly during the month of liver fibrosis induction to monitor their growth rate. The number of rats in each group at the beginning and end of the experiment was also recorded, and mortality percent was determined.

### Serum biochemical analysis

Twenty four hours following the last TAA injection, blood samples were withdrawn from six rats per group via retro-orbital vein under light ether anesthesia [27]. Serum was used for estimation of albumin, alkaline phosphatase (ALP) and bilirubin levels, using specific diagnostic kits (Biodiagnostic, Egypt).

### Tissue biochemical analysis

Immediately after blood sampling, animals were sacrificed by cervical dislocation under ether anesthesia, livers were rapidly removed, washed in ice-cooled saline and plotted dry. A weighed part of each liver was homogenized, and the homogenate was used for assessment of; reduced glutathione (GSH) and lipid peroxides measured as malondialdehyde (MDA) contents using (Biodiagnostic, Egypt) kits, nuclear factor kappa B (NF-κB) and alpha-fetoprotein (AFP) contents using specific diagnostic kits (Glory Science, USA), as well as hydroxyproline (HP) content according to Woessner assay [28].

### Histopathological and Immunohistochemical examination

Specimens from three major lobes of the liver tissue were dissected immediately after scarification, formalin-fixed and paraffin-embedded according to Carleton (1980) [29]. For histopathological investigation, serial sections of 6 μm thick were cut and stained with Haematoxylin and Eosin (H&E), Masson Trichrome (MT) stains. Additionally, apoptotic cells in liver tissue sections were determined immunohistochemically by Terminal deoxy Uridine triphosphate Nick End Labeling (TUNEL) assay [30]. Apoptotic cells were identified by a brown stain over the nuclei. All sections were scanned and analyzed, and images were captured and processed using Adobe Photoshop version 8.0.

### Statistical analysis

Results were expressed as mean ± standard error (SE) of the mean. Data were statistically analyzed using Stat-graphics Centurion ΧV version 15.2.06, Stat point, Inc. Data of rat body weights were analyzed using repeated measures two-way analysis of variance (ANOVA) to test for interaction between drug type, time and body weight followed by least significant difference (LSD) multiple range test. All other data were statistically analyzed using One-way ANOVA followed by LSD multiple range test. For all data, a probability of less than 0.05 was used as criterion for statistical significance.

## Results

### Body weight and mortality percent

**Figure 1:**
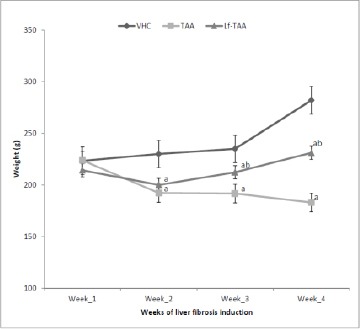
*Rats body weight*. VHC, rats treated with vehicles; TAA, rats treated with thioacetamide; Lf-TAA, rats treated with lactoferrin and thioacetamide. ^a^Significantly different from VHC at respective time; P < 0.05. ^b^Significantly different from TAA at respective time; P < 0.05.

Body weights of VHC-treated rats were increased by 26% throughout the month of TAA-induced liver fibrosis, while they were decreased by 18% in TAA group. In Lf-TAA-treated animals, body weights were increased up to 108% (Fig. 1). No mortality was observed in VHC group, while it reached 67% and 25% in TAA and Lf-TAA groups, respectively.

### Serum parameters

TAA-induced liver fibrosis rat model markedly decreased normal serum albumin and increased serum ALP and bilirubin levels. In Lf-TAA-treated rats, normal serum albumin and ALP levels were preserved. Though, Lf failed to alter TAA-induced increase of serum bilirubin level (Table 1).

**Table 1:** Serum albumin, alkaline phosphatase and bilirubin levels.

Parameters Groups	Albumin (g/dl)	ALP (IU/l)	Bilirubin (mg/dl)
**VHC**	3.82^b^± 0.24	109.25^b^± 3.96	0.70^b^± 0.07
**TAA**	3.09^a^± 0.16	116.03^a^± 0.52	1.01^a^± 0.02
**Lf-TAA**	3.70^b^± 0.28	113.16 ± 0.22	1.07^a^± 0.04

VEH, rats treated with vehicles; TAA, rats treated with thioacetamide; Lf-TAA, rats treated with lactoferrin and thioacetamide; ALP, Alkaline phosphatase. Data are presented as mean ± SE, n=6.

a Significantly different from VHC; P< 0.05.

b Significantly different from TAA; P< 0.05.

### Tissue parameters

Normal liver MDA content was increased in TAA-liver fibrosis rat model to 141% and GSH was decreased to 30%. Furthermore, TAA increased normal liver contents of HP, NF-κB and AFP to 201%, 198% and 153%, respectively. In Lf-TAA group, normal liver contents of all measured tissue parameters were conserved (Table 2).

**Table 2:** Liver contents of malondialdehyde, reduced glutathione, hydroxyloroline, nuclear factor kappa B and alpha fetoprotein.

Parameters Groups	MDA (mmol/g)	GSH (mmol/g)	HP (µg/g)	NF-κB (ng/g)	AFP (ng/g)
**VHC**	149.36^b^ ± 10.48	8.13^b^ ± 0.43	25.52^b^ ± 1.09	7.01^b^ ± 0.54	8.21^b^ ± 0.43
**TAA**	210.20^a^ ± 11.13	2.43^a^ ± 0.20	51.59^a^ ± 3.78	13.88^a^ ± 1.19	12.55^a^ ± 0.63
**Lf-TAA**	149.59^b^ ± 13.71	8.29^b^ ± 0.43	30.91^b^ ± 1.85	8.56^b^ ± 0.65	8.56^b^ ± 0.68

VEH, rats treated with vehicles; TAA, rats treated with thioacetamide; Lf-TAA, rats treated with lactoferrin and thioacetamide; MDA, Malondialdehyde; GSH, Reduced glutathione; HP, Hydroxyproline; NF-κ B, Nuclear factor kappa B; AFP, Alpha fetoprotein. Data are presented as mean ± SE, n = 6.

a Significantly different from VHC; P < 0.05.

b Significantly different from TAA; P < 0.05.

### Histopathological and immunohistochemical features (Fig. 2, 3, and 4)

**Figure 2:**
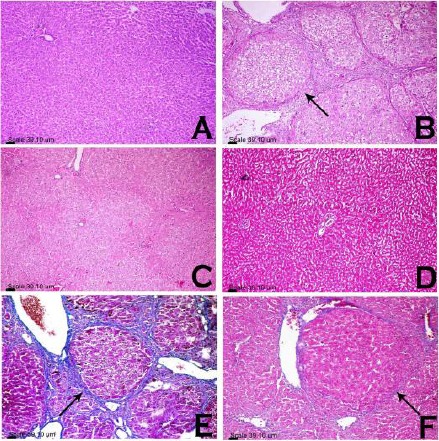
*Photomicrograph of rats hepatic lobules. Liver sections from rats treated with VHC (A, D), TAA (B, E) and Lf-TAA (C, F), stained with H&E stain (A, B, C) and MT stain (D, E, F). Histopatological examination showed (A) normal hepatic architecture and preserved lobular pattern, (b) hepatic fibrosis and extensive fibroblastic proliferation (arrow), (C) preserved lobular pattern, (D) no evidence of fibrosis or inflammatory reaction in hepatic lobules, (E) blue-stained fibrous connective tissue (arrow) and (F) very delicate blue-stained fibrous tissue (arrow) encircling hepatic lobules*.

**Figure 3:**
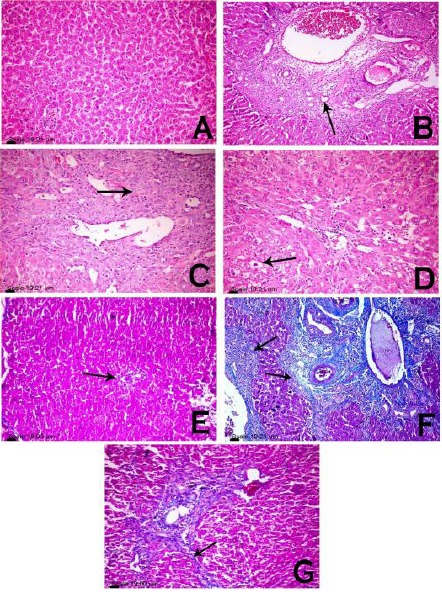
*photomicrograph of rats portal triad. Liver sections from rats treated with VHC (A, E), TAA (B, C, F) and Lf-TAA (D, G), stained with H&E stain (A, B, C, D) and MT stain (E, F, G). Histopatological examination showed (A) normal portal area, (B) dilatation and congestion of portal blood vessels, portal fibrosis, hyperplasia of biliary epithelium and formation of newly formed bile ductules (arrow), (C) intense fibroblastic proliferation (arrow) mixed with oval cells and biliary cyst formation, (D) normal portal area with few mononuclear cell infiltration associated with vacuolar degeneration of the surrounding hepatocytes (arrow), (E) normal portal area (arrow), (F) blue stained fibrous connective tissue expanding portal area (arrow) and (G) portal area less expanded by blue stained fibrous connective tissue (arrow)*.

**Figure 4:**
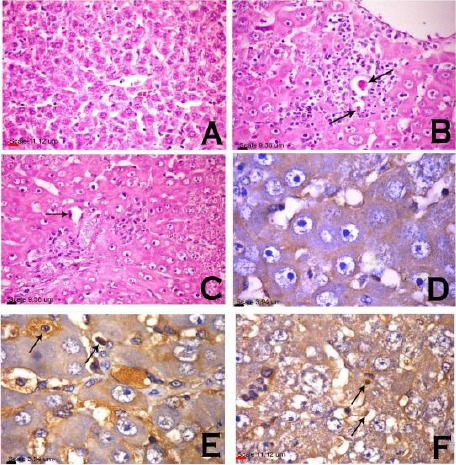
*Histopathological and immunohistochemical analysis of apoptosis. Liver sections from rats treated with VHC (A, D), TAA (B, E) and Lf-TAA (C, F), stained with H&E stain for histopathological examination (A, B, C) and immunohistochemically analysed for cleaved caspase-3-positive hepatocytes (D, E, F), showed (A) no evidence of necrosis or apoptosis, (B) focal area of hepatocellular necrosis infiltrated with mononuclear cells with presence of apoptotic cells (arrow), (C) apoptotic cells (arrow), (D) cleaved caspase-3-negative hepatocytes, (E) pre-apoptotic hepatocytes characterized by a positive antibody-reaction with cytoplasmic (long arrow) and nuclear localization (short arrow) and (F) cleaved caspase-3-positive hepatocytes with cytoplasmic (long arrow) and nuclear (short arrow) staining*.

Compared to VHC group, liver sections of TAA-treated rats revealed disorganized lobular pattern with hepatic fibrosis and extensive fibroblastic proliferation. Portal area revealed marked dilatation and congestion of portal vessels, portal fibrosis, hyperplasia of biliary epithelium and formation of newly formed bile ductules. Intense fibroblastic proliferation, extended to infiltrate the surrounding hepatic parenchyma, mixed with oval cells and biliary cyst formation, were also demonstrated.

Hepatocytes showed hypertrophy with large karyomegallic nuclei and prominent nucleoli associated with focal area of necrosis infiltrated with mononuclear cells with presence of few apoptotic cells. Immunohistochemical demonstration of cleaved caspase-3-positive hepatocytes in which putative pre-apoptotic hepatocytes could be identified, was characterized by a positive antibody-reaction with cytoplasmic and nuclear localization.

In Lf-TAA group, less inflammatory changes were observed with preserved lobular pattern and very delicate deposition of fibrous tissue in hepatic lobules and portal area. Portal infiltration with few mononuclear cells associated with vacuolar degeneration of the surrounding hepatocytes, was also observed. Hepatocytes were greatly similar to those of VHC group; however, presence of apoptotic cells was demonstrated. Immunohistochemical-stained cleaved caspase-3-positive hepatocytes showed more positive antibody-reacting hepatocytes compared to TAA group.

## Discussion

Induction of liver fibrosis through TAA caused marked toxicity in rats revealed by their inability to gain body weight. Previous studies attributed the same outcome to lower levels of nutrient absorption and metabolic efficiency after exposure to TAA [31, 32]. In Lf-TAA group, weight gain was nearly parallel to those in normal group. This suggests that Lf diminished the negative effect of TAA on growth rate [26].

Mortality rate observed in TAA group is corresponding with many previous studies reported elevated mortality rate within rats with TAA-induced liver fibrosis [32, 33]. In Lf-TAA rats, mortality rate was reduced in agreement with another study found Lf to reduce mortality rate in mice with acetaminophen-induced liver injury [21].

Albumin is a plasma protein that is synthesized solely in liver and its serum level correlates with hepatocellular synthesis capacity [34]. The current hypoalbunimea in TAA-treated rats reflects liver impaired ability. Previous studies [35, 36] contributed the same finding to extensive hepatocytes damage and alterations in their synthetic mechanisms by TAA-hepatotoxic metabolites [36]. Lf significantly prevented this hypoalbunimea which reflects the maintenance of normal hepatocellular synthesis capacity. Similarly, other studies showed that Lf stimulates kupffer cells to produce cytoprotective mediators [21] and it can retain the normal ability of hepatocytes to synthesize albumin [37].

Correspondingly, the current elevated serum activity of ALP in TAA group is accounted to hepatic parenchymal damage with subsequent diminished liver function capacity that lead to elevated serum activity of liver enzymes than normal; including ALP [38], a result consistent with the work of Galisteo et al [39]. Lf in the current experiment maintained normal serum ALP activity. These results are consistent with results of previous studies [40, 41].

Serum bilirubin had been markedly elevated in TAA-treated rats, a result in agreement with previous studies [2, 42]. Physiologically, bilirubin is a haem product that undergoes catalysis and conjugation with glucuronic acid in hepatocytes before being excreted into bile. Hyperbilirubinemia indicates liver failure to conjugate and excrete bilirubin [2]. Lf failed to maintain normal bilirubin serum level in this study, a result against that of earlier study [26] in which Lf restored normal bilirubin level in rat model of colitis. The proposed explanation of this controversy is that the earlier study used Lf for double the duration used in the current study and according to Al-Jumaily and Khaleel [43]; prolonged time is needed to restore normal serum bilirubin level.

Marked oxidative stress was observed in current TAA liver fibrosis rat model, evidenced by rise in liver content of lipid peroxidation end product, MDA, and decline in GSH content. Obtained results are in agreement with other studies [44, 45] and attributed to TAA biotransformation with extensive production of reactive oxygen species (ROS) exceeding the capacity of endogenous antioxidant protective mechanisms; including GSH, to eliminate them, and in turn, GSH depletion [46]. Under influence of these ROS, MDA are produced from oxidation of polyunsaturated fatty acids in biomembranes [47]. Lf markedly prevented this TAA-induced oxidative stress, prevented MDA accumulation and GSH depletion. Consistently, other studies showed antioxidant effect of Lf [48-50].

HP is a major component of collagen that formulates the highest share of ECM deposits, and so, considered as a sensitive marker of fibrosis [25]. In the present TAA group, liver HP content was increased. Similarly, several studies reported significant elevation in HP and collagen contents in rats subjected to TAA intoxication [25, 36, 44]. Additionally, several studies strongly implicated NF-κB as a potential master orchestrator in liver fibrosis [51, 52]. After activation with pro-inflammatory cytokines; NF-κB becomes the driving force of fibrosis. It activates the major fibrogenic molecule; tissue growth factor beta (TGF-β), and stimulates the survival and production of activated myofibroblasts through differentiation of HSCs [53]. NF-κB-liver content was increased in the present TAA liver fibrosis model in correspondence with another study [54]. In Lf-TAA-treated rats, normal tissue contents of both HP and NF-κB were preserved, a result in harmony with that of Togawa et al [26] and other studies postulated that attenuation of NF-κB plays an important role in fundamental block of developing fibrosis and subsequently, decreases tissue HP content [48, 55].

AFP is often measured as a tumor marker for diagnosing hepatocellular carcinoma (HCC). However, recent studies postulated that AFP can be used in diagnosis and assessment of liver fibrosis without the presence of HCC [10, 56]. Currently, a Marked increase in liver content of AFP was observed in TAA group in consistence with another study [57], while, its normal liver content was maintained in Lf-TAA group. This result reflects the hepatoprotective ability of Lf against liver fibrosis induced by TAA and supported by the work of Harn et al [10] showed maintenance of AFP content in liver to be an indicator of protection against fibrosis.

The current histopathological and immuno-histochemical manifestations seem to be in accordance with the present biochemical findings. Liver fibrosis revealed in TAA group is parallel with the findings of previous studies [2, 58], and preservation of normal liver architecture with limited collagen deposition in Lf-TAA group is consistent with other studies [21, 59]. The low rate of apoptosis reported immunohistochemically in TAA group is in accordance with former study found that TAA injection caused mainly necrosis along with minimal apoptosis [31]. The high rate of apoptosis detected in Lf-TAA group is supported by the results of previous [23] in which Lf inhibited growth factors, arrested cell growth and stimulated kupffer and NK cells to exert their apoptotic actions. This suggests pro-apoptotic actions of Lf favored activated-myofibroblasts apoptosis and in turn resolution of liver fibrosis.

Based on all the findings, it could be concluded that treatment of rats with Lf (200 mg/kg/day, po), for one month before and another month during TAA intoxication, exhibited marked hepatoprotective effect evidenced by biochemical results and proven by histopathological and immunohistochemical examinations. These findings revealed that this hepatoprotective potential is possibly through its antioxidant and pro-apoptotic actions, as well as, through its inhibitory effect on NF-κB.

This study strongly suggests Lf as a promising drug for protection against structural and functional changes associated with liver fibrosis. Additionally, because Lf has been found to show an antiviral activity against HCV [14, 60, 61]; one of the most important causative factors of liver fibrosis, the current findings suggest additional value of using Lf as a hepatoprotective therapy in HCV patients. Moreover, many recent studies revealed that Lf could be used as a targeting system to deliver drugs to liver as it can bind to multiple receptors on hepatocytes [62]. The findings of the present study add an advantage to the use of such delivery system in patients with, or susceptible to, liver fibrosis.

**Abbreviations:**
Lf, Lactoferrin
TAA, Thioacetamide
ip, Intraperitoneal
p.o., oral
VHC, vehicle
ALP, Alkaline phosphatase
GSH, Reduced glutathione
MDA, Malondialdehyde
HP, Hydroxyproline
NF-κB, Nuclear factor kappa B
AFP, Alpha fetoprotein
ECM, Extracellular matrix
HSCs, Hepatic stellate cells
HCV, Hepatitis C virus
NK, Natural killer
NRC, National Research Center
VHC group, normal group
TAA group, liver fibrosis-model group
Lf-TAA group, Lf-treated group
H&E, Haematoxylin and Eosin stain
MT, Masson Trichrome stain
TUNEL, Terminal deoxy Uridine triphosphate Nick End Labeling
SE, standard error
ANOVA, analysis of variance
LSD, least significant difference
ROS, reactive oxygen species
TGF-β, tissue growth factor beta
HCC, hepatocellular carcinoma.
